# Chronic obstructive pulmonary disease (COPD): neutrophils, macrophages and lymphocytes in patients with anterior tuberculosis compared to tobacco related COPD

**DOI:** 10.1186/s13104-018-3309-6

**Published:** 2018-03-27

**Authors:** Elise Guiedem, George Mondinde Ikomey, Céline Nkenfou, Pefura-Yone Eric Walter, Martha Mesembe, Novel Njweipi Chegou, Graeme Brendon Jacobs, Marie Claire Okomo Assoumou

**Affiliations:** 1 Center for the Study and Control of Communicable Diseases (CSCCD), Faculty of Medicine and Biomedical Sciences, University of Yaounde 1, Yaoundé, Cameroon; 2 Chantal BIYA International Reference Centre for Research on HIV/AIDS Prevention and Management (CBIRC), Yaoundé, Cameroon; 3 Pneumological Service, Yaoundé Jamot Hospital, Yaoundé, Cameroon; 4 DST/NRF Centre of Excellence for Biomedical Tuberculosis Research and SAMRC Centre for Tuberculosis Research, Division of Molecular Biology and Human Genetics, Department of Biomedical Sciences, Faculty of Medicine and Health Sciences, Stellenbosch University, PO Box 241, Cape Town, 8000 South Africa

**Keywords:** COPD, Tuberculosis, Tobacco, Neutrophils, Monocytes, Lymphocytes

## Abstract

**Objective:**

The inflammatory profile of chronic obstructive pulmonary disease (COPD) related to tobacco is known in certain studies while that of the post tuberculosis form is not yet known. This study aimed to evaluate the levels of neutrophils, macrophages and lymphocytes cells in sputum of COPD patients with history of smoking or anterior tuberculosis. Enumeration of cells in samples was analyzed using standard microscopy.

**Results:**

We enrolled 92 participants, 46 (50%) were COPD subjects comprising 22 (47.83%) smokers and 24 (52.17%) with anterior tuberculosis while 46 (50%) healthy persons constituted the control group. The levels of neutrophils, lymphocytes and monocytes were statistically higher in COPD patients compared to the control group with p-values of 0.0001 respectively. Neutrophils levels were higher in COPD patients with history of tobacco than in COPD patients with anterior tuberculosis with a mean rate of 4.72 × 10^6^/ml and 2.48 × 10^6^/ml respectively (p = 0.04). The monocytes and lymphocytes levels were not statistically different between the two sub-groups of COPD patients with p-value of 0.052 and 0.91 respectively. Neutrophils are the only inflammatory cells that were significantly higher in COPD patients with history of smoking as compared to COPD patients with anterior tuberculosis.

## Introduction

Chronic obstructive pulmonary disease (COPD) is a disease affecting respiratory airways combining emphysema and chronic bronchitis [1]. According to the global initiative for obstructive lung diseases (GOLD), COPD will rise to the third most cause of death worldwide by 2020. The diagnosis is based on clinical assessment and spirometric data including: forced expiratory volume (FEV), forced vital capacity (FVC), and FEV/FVC ratio. COPD is defined by the presence of irreversible obstruction ventilator trouble (OVT). Irreversible OVT is defined by a FEV/FVC ratio lower than 70% and the absence of a complete reversibility after inhalation of 400 μg of salbutamol [2]. The severity of COPD is evaluated based on the stages of GOLD: stage I, FEV > 80%; stage II, FEV between 50–80%; stage III, FEV between 30–50%; stage IV, FEV < 30% [3].

COPD is mainly caused by smoking and the inflammatory profile is known in developed countries [4]. The defense system of the respiratory airways consists of the mucociliary carpet and epithelial cells tight junctions which will be broken by chronic exposure to cigarette smoke that aggress it causing epithelial damage [5, 6]. These lesions linked to intoxication cause a local infiltration of neutrophiles, macrophage, and lymphocytes that release the mediators which act on the airways walls and induce fibrosis, increased muscle mass and airway wall thickness, narrowing of airways, and causing limitation of respiratory flow [7]. These damages induce emphysema and chronic bronchitis that are peculiar of COPD [8, 9].

Although smoking is the main cause of COPD [10], anterior tuberculosis can also induce the disease. During tuberculosis, inflammation of the bronchial endothelium induces: bronchial obstruction, pulmonary fibrosis, edema of airway mucosa, hypertrophy of the submucosal glands, which leads to airway limitation, main characteristics of COPD [11, 12]; however, the inflammatory profile of COPD in patients with history of tuberculosis is not yet known.

The use of anti-inflammatory drugs in the management of COPD may require knowledge of the inflammatory profile to adapt the treatment. The aim of this study was to evaluate the levels of neutrophils, macrophages and lymphocytes in COPD patients with history of smoking as compared to those with history of tuberculosis.

## Main text

### Methods

Participants were enrolled from March 2016 to April 2017 at the Yaoundé Jamot Hospital (YJH); the main and biggest reference centre specialized in the management of pulmonary diseases. Participants comprised COPD patients and healthy participants that served as control. Age and sex of COPD patients were matched to controls. COPD participants were patients consulting at the YJH and diagnosed by a pneumologist, comprising two sub-groups: COPD patients with a history of smoking (COPD/post tobacco) and COPD with a history of tuberculosis (COPD/post Tb). Sputum samples were collected from each participant and analyzed at the Center for the Study and Control of Communicable Diseases (CSCCD) of the Faculty of Medicine and Biomedical Sciences, University of Yaoundé I.

Once the pneumologist diagnosed a patient with COPD fulfilling the inclusion criteria, the spirometric results of these patients were extracted from their medical record. The history of tuberculosis or smoking was also extracted from their medical record and confirmed by each patient.

Spirometric measurements for the control group was done with the turbine pneumotachograph (care fusion) following the American Thoracic Society (ATS) standard of 2010 by an experienced nurse specialized in respiratory diseases and the results interpreted by the pneumologist to ensure that these people do not have any respiratory problems.

Sputum was collected in a sterile container for neutrophils, macrophages and lymphocytes counts. Cell counts were performed after staining of the smear with May–Grunwald Giemsa (MGG) and read by standard microscopy.

This work was approved by the Cameroon National Ethical Committee of Research for Human Health (No 2016/06/772/CE/CNERSH/SP). All participants gave their verbal and signed consent after receiving detailed information on the study.

Data for the study was entered into Microsoft Excel sheet and were analyzed with GraphPad PRISM version 5.0 software (GraphPad Software, Inc., La Jolla, California, USA). Chi square test was used to run statistical analysis for binary variables. Student t-test and its non parametric equivalent were used to compare means or median. p-values below 0.05 were considered statistically significant with a confidence interval (CI) of 95%.

### Results

#### Demographic characteristics

A total of 92 participants comprising 42 (45%) male and 50 (55%) female were included. Participants’ age ranged from 25 to 80 years. The enrollment consisted of 46 COPD patients and 46 healthy persons for the control group. These 46 COPD patients comprised two sub-groups: 22 COPD/post tobacco patients made up of 20 (91%) males and 2 (9%) females, with a mean age of 63 ± 10.45 years, and 24 COPD/post tuberculosis (Tb) patients made up of 8 (33.3%) males and 16 (66.7%) females with a mean age of 43 ± 12.38 years. A statistically significant difference was noted between the two COPD sub-groups with respect to sex and age with p-values of 0.0023 and 0.0002 respectively.

#### Clinical characteristics

##### Comparison of FEV in the two sub-groups of COPD patients

In COPD/post tobacco patients, the FEV ranged from 20.3 to 62.60% with an average of 36.88% (± 14.95%). For COPD/post Tb, the FEV varied from 30 to 79% with an average of 53.30% (± 17.21%). The FEV was lower in COPD/post tobacco patients than in COPD/post Tb sub-group, p = 0.015.

##### Comparison of FEV/FVC ratio in two sub-groups of COPD patients

In COPD/post tobacco patients, the FEV/FVC ratio ranged from 36 to 68% with an average of 50.54% (± 12.09%). The FEV/FVC ratio of the COPD/post Tb varied from 36 to 72.35% with an average of 62.79% (± 17.95%). The FEV/FVC was lower in COPD/post tobacco than the COPD/post Tb sub-group, p = 0.033.

##### Comparison of COPD severity in patients (GOLD classification)

In COPD/post tobacco patients, the stages were more advanced compare to COPD/post Tb. COPD/post tobacco sub-group comprised 11 (50%) stage IV, 6 (27.3%) stage III and 5 (22.7%) stage II. COPD/post Tb comprised 3 (12.5%) stage IV, 9 (37.5%) stage III and 12 (50%) stage II. A statistically significant difference was noted between the two sub-groups (p = 0.032).

#### Biological analysis of sputum

##### Determination of neutrophils rates in COPD and control

**Fig. 1:**
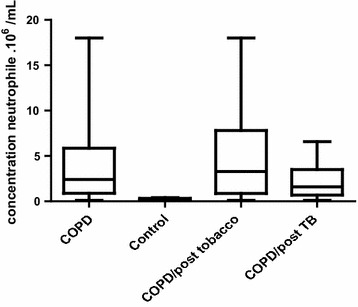
Concentration of neutrophiles in COPD patients and in control group

The neutrophils rate (3.57 × 10^6^ ± 3.8 × 10^6^/ml) was statistically higher in COPD patients as compared to controls (0.3 × 10^6^ ± 0.12 × 10^6^/ml) with p = 0.0001 (Fig. 1).

In the COPD/post tobacco sub-group, the neutrophils level (4.72 × 10^6^ ± 4.89 × 10^6^/ml) was statistically higher than those of COPD/post Tb (mean rate: 2.48 × 10^6^ ± 2.29 × 10^6^/ml), p = 0.04 (Fig. 1).

##### Determination of lymphocyte rate in COPD and control

**Fig. 2:**
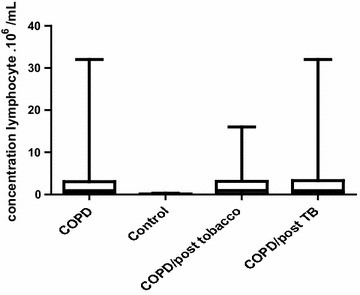
Concentration of lymphocytes in COPD patients and in control group

The lymphocytes level of 3.38 × 10^6^ ± 6.5 × 10^6^/ml was higher in the COPD patients compared to those of control which was 0.10 × 10^6^ ± 0.1 × 10^6^/ml, p = 0.0001 (Fig. 2).

In COPD/post tobacco patients, the lymphocytes rate (2.81 × 10^6^ ± 4.4 × 10^6^/ml) was not statistically different compared to those of COPD/post Tb (3.91 × 10^6^ ± 8.07 × 10^6^/ml), p = 0.91 (Fig. 2).

##### Determination of monocytes counts in COPD and control

**Fig. 3:**
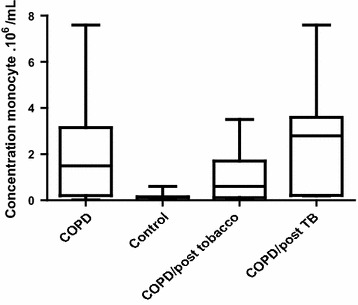
Concentration of monocytes in COPD patients and in control group

The monocytes count was statistically higher in COPD patients (1.90 × 10^6^ ± 2.03 × 10^6^/ml) compare to the control group (0.14 × 10^6^ ± 0.097 × 10^6^/ml), p = 0.0001 (Fig. 3).

There was no statistically significant difference between the two COPD sub-group based on monocytes counts, p = 0.052 (Fig. 3).

### Discussion

With respect to sex, males were predominant in the COPD/post tobacco sub-group compare to COPD/post Tb patients (p-value: 0.0023); suggesting that culturally fewer women actively smoked in the Cameroonian society.

COPD/post tobacco patients were older than COPD/post Tb group (p = 0.0002). In several cases, tobacco smoking begins when people are already adult, and the airway tract is healthy. It is after several years of exposure to cigarette smoke that COPD is established [13, 14]. While tuberculosis can be contracted at any age (even childhood), which induces abnormal functioning of the airway tract especially obstructive airways complications [15, 16] which can cause a rapid development of COPD.

The FEV percentage and FEV/FVC ratio were lower in COPD/post tobacco than in COPD/post Tb; consequently, the COPD/post tobacco sub-group had COPD stages more severe than the stages of COPD/post Tb sub-group. These results suggest that the perturbations and irritations caused by smoking are more pronounced than those induced by tuberculosis. Antituberculosis drugs kill mycobacterium and thus reduce the complications due to mycobacterium itself, whereas with smokers, cigarette smoking continues to damage until the establishment of COPD.

In sputum, the high levels of neutrophils, lymphocytes and monocytes in COPD patients when compared to the control group suggest that cellular inflammation in the airway tract is important during COPD. These results are comparable with other studies such as Gibson et al., Casio et al. and Profita et al. who found high levels of these cells in the airway tract of COPD patients compared to clinically healthy persons.

However, by comparing the two sub-groups of COPD patients, neutrophils rate were higher in COPD/post tobacco than COPD/post Tb (p = 0.04), indicating that neutrophils are more involved in COPD related to tobacco. Indeed, neutrophils are the main cellular marker of innate immunity during cigarette smoking because, they infiltrate into smokers airway tract before advancement to COPD [17]. Neutrophils are attracted into the airway tract by the intoxication induced by cigarette smoking. Neutrophils secrete substances like 5-hydroxy-eicosanotetraenoic acid, Leukotriene B4 (which contribute to the chemotactic activity of expectorations), myeloperoxidase (which activates the secretion of interleukin-8), elastase (which cases the elasticity loss of elastic fiber and promote epithelial cells to produce more IL-8 and Leukotriene B4). Neutrophils also produce IL-1 which contributes in leukocytosis activity and in the production of other cytokines [17, 18].

The levels of monocytes and lymphocytes were similar in the two groups of COPD patients with p-value of 0.052 and 0.91 respectively suggesting that these cells act at the same level in both forms of COPD.

Monocytes/macrophages in COPD caused by tobacco smoking produce proteins of the extracellular matrix, lipid mediators, Leukotriene, prostaglandines, cytokines, chemokines and the metalloproteinase matrix (MMP), these substances are associated in both fibrosis of the small airways and to centrilobular emphysema in COPD. In culture, monocytes of smokers and those of COPD patients release an important rate of MMP-1 and 9, IL-6 and the monocyte chemotactic protein-1 (MCP-1) [18, 19].

Looking at lymphocytes, CD8 T-lymphocytes play an important role in inflammation and the development of emphysema by producing Interferon gamma (IFN- ϒ), interferon-inducible protein-10 (IP-10) and monokine induced by interferon-gamma (MIG). These products promote the secretion of MMP and chemokine (IL-8 and MCP-1) by macrophages [20]. The actions of these substances on the endothelial cells make them change and remodel their membrane favoring the macromolecules lost and cellular extravasations. CD8 T lymphocytes also have a role in the development of COPD because the cytotoxic activity of CD8 is more pronounced due to the secretion of perforin and granzymes which lyses pulmonary parenchyma cells [21]. CD8 lymphocyte also expresses as which lead to apoptosis of the epithelial and endothelial cell. The principal function of CD8 cells is to eliminate infected cells by cytolysis or by apoptosis [22].

B cells in COPD generally reflect both an adaptive response against chronic infections in the advanced COPD or as auto-immune response originally induced by auto-antibodies [21]. Approximately 70% of COPD patients have Ig-G auto-antibodies circulating against epithelial cell [21]. The auto-immune mechanism in chronic inflammation and emphysematous damage are justified by the detection of circulating auto-antibodies against undamaged proteins. These auto-antibodies are mainly anti-elastin and anti-epithelial in smokers COPD. Contrarily to COPD related to tobacco, studies on COPD caused by tuberculosis have shown several risks conducive to COPD [23, 24] but, have not decrypted the relation between the pathogenesis of the diseases and the inflammation.

### Conclusion

We therefore conclude that the pathogenesis of COPD/post Tb could be associated with pulmonary neutrophilic polynucleosis, monocytosis and lymphocytosis same as in COPD/post tobacco, with a higher level of neutrophils in COPD/post tobacco.

### Limitation

The main strength of this study was that it was done in a resource limited setting in which the prevalence of Tb is high with an important incidence of post Tb pulmonary obstruction where limited studies have been carried out. A limit of this study was that we did not distinguish the levels of T lymphocytes from B lymphocytes.

## Abbreviations

ATS: American Thoracic Society
CD: cluster differentiation
CI: confidence interval
COPD: chronic obstructive pulmonary disease
CSCCD: Center for the Study and Control of Communicable Diseases
FEV: forced expiratory volume
FVC: forced vital capacity
GOLD: global initiative for obstructive lung diseases
IFN: interferon
Ig: immunoglobuline
IL: interleukin
IP: interferon-inducible protein
MCP: monocyte chemotactic protein
MGG: May–Grunwald Giemsa
MIG: monokine induced by interferon-gamma
ml: milliliter
MMP: metalloproteinase matrix
OVT: obstruction ventilator trouble
Tb: tuberculosis
YJH: Yaoundé Jamot Hospital
